# Measuring behavioral responses of sea turtles, saltwater crocodiles, and crested terns to drone disturbance to define ethical operating thresholds

**DOI:** 10.1371/journal.pone.0194460

**Published:** 2018-03-21

**Authors:** Elizabeth Bevan, Scott Whiting, Tony Tucker, Michael Guinea, Andrew Raith, Ryan Douglas

**Affiliations:** 1 Department of Biology, University of Alabama at Birmingham, Birmingham, Alabama, United States of America; 2 Department of Biodiversity, Conservation and Attractions, Parks and Wildlife Service-Marine Science Program, Kensington, Western Australia, Australia; 3 AusTurtle, Inc., Charles Darwin University, Darwin, Northern Territory, Australia; Florida State University, UNITED STATES

## Abstract

Drones are being increasingly used in innovative ways to enhance environmental research and conservation. Despite their widespread use for wildlife studies, there are few scientifically justified guidelines that provide minimum distances at which wildlife can be approached to minimize visual and auditory disturbance. These distances are essential to ensure that behavioral and survey data have no observer bias and form the basis of requirements for animal ethics and scientific permit approvals. In the present study, we documented the behaviors of three species of sea turtle (green turtles, *Chelonia mydas*, flatback turtles, *Natator depressus*, hawksbill turtles, *Eretmochelys imbricata*), saltwater crocodiles (*Crocodylus porosus)*, and crested terns (*Thalasseus bergii*) in response to a small commercially available (1.4 kg) multirotor drone flown in Northern Territory and Western Australia. Sea turtles in nearshore waters off nesting beaches or in foraging habitats exhibited no evasive behaviors (e.g. rapid diving) in response to the drone at or above 20–30 m altitude, and at or above 10 m altitude for juvenile green and hawksbill turtles foraging on shallow, algae-covered reefs. Adult female flatback sea turtles were not deterred by drones flying forward or stationary at 10 m altitude when crawling up the beach to nest or digging a body pit or egg chamber. In contrast, flyovers elicited a range of behaviors from crocodiles, including minor, lateral head movements, fleeing, or complete submergence when a drone was present below 50 m altitude. Similarly, a colony of crested terns resting on a sand-bank displayed disturbance behaviors (e.g. flight response) when a drone was flown below 60 m altitude. The current study demonstrates a variety of behavioral disturbance thresholds for diverse species and should be considered when establishing operating conditions for drones in behavioral and conservation studies.

## Introduction

Unmanned aerial vehicles (i.e. UAVs or drones) have become widely-used as a cost-effective tool in wildlife conservation, management, and research. Drones are an efficient method for evaluating animal behavior, abundance and distribution [1–3]; enhancing animal photo ID and photogrammetry [1, 4–6]; and increasing the accuracy of data collection relative to traditional methods (e.g. surveys conducted on foot, by boat, or by terrestrial vehicle) [6–8]. Additionally, a growing network of drone operators, hobbyists, and commercial users is broadening access to an array of open source image processing and operating platforms. A key benefit of using drones in wildlife studies is to minimize the potential influence of observer presence. Many applications (e.g. identifying the species, sex, and conducting population censuses, measuring abiotic and biotic factors, and observing the behavior of one or a small group of distinct subjects or densely aggregated individuals [9–12]) require the operation of drones (typically multirotor) in close proximity to target species (e.g. <30 meters) to achieve sufficient resolution. However, studies focusing on the effects of drones near wildlife are limited [4]. To evaluate the impact of drones on target species requires knowledge of: 1) the capabilities of a target species to detect the drone, 2) the nature of disturbance introduced by a specific model drone, and 3) background conditions of the habitat in which the study is conducted (e.g. ambient noise levels). Only then can an attempt be made to understand the broader implications of applying drone technology to behavioral and ecological studies that enhance wildlife conservation, research, and management.

Drones have been used to study a suite of terrestrial [1, 12–17] and marine species [3, 5, 8, 18, 19], including elephants [20, 21], cetaceans [18, 22, 23], and sea turtles [9, 10, 24, 25]. Despite the increasing applications for drones in wildlife biology [11, 26–30], relatively few studies specifically focus on assessing the behavioral responses of taxa to drones at low altitudes. Some key exceptions [5, 12, 15, 31, 32] highlight the complexity of this endeavor.

A critical component for evaluating the level of behavioral disturbance imposed by drones is to understand the spectrum of responses displayed by each species. Sea turtles in shallow habitats (i.e. <1 m water depth) can detect and respond to a threatening stimulus (e.g. humans walking towards them in shallow water) by swimming at high speed towards deeper water, often generating a “bow wave” in front of the turtle (E. Bevan, *pers*. *obs*.). In deeper water habitats (i.e. >1 m water depth) acoustic disturbances to sea turtles (e.g. high-pressure air gun pulses and nearby vessels or objects) can elicit a range of behavioral responses from head retraction, flipper movement, and changes in swimming patterns and orientation [33], to diving [34–36]. A range of behavioral responses to auditory disturbance have been reported in birds, including crested terns (*Thalasseus bergii)*, ranging from minor head-scanning to flushing [7, 37, 38]. Pomeroy et al. [5] examined two species of pinnipeds and found that behavioral responses (varying from increased alertness to fleeing towards the water) varied depending on the type of drone used, and age, sex, and in some cases, reproductive status of individuals. Such studies suggest that future research using drones should consider these variables, in addition to altitude when assessing threshold levels for behavioral responses to drones.

Evaluating drone detectability at different altitudes is multifaceted and involves assessing the sensory abilities of target species to discern the drone or its shadow (visual component) and detect the unique noise emission characteristics of each drone (auditory component). Although limited, there are some data available on the auditory capabilities of sea turtles, crocodilians, and shorebirds that provide a basis for understanding whether these species can detect the sound emitted by drones and at what specific threshold. In sea turtles (loggerhead (*Caretta caretta*) and *C*. *mydas*), peak auditory sensitivity in air occurs between 300 and 400 Hz, and in water between 50 and 400 Hz [39, 40]. The American alligator (*Alligator mississipiensis*) exhibits a range of auditory sensitivity in water from 100 Hz to 2 kHz, and in air 100 Hz to 8 kHz, with peak sensitivities in water at 800 Hz and in air at 1000 Hz [41]. Audiograms from 49 species of birds suggest that birds generally exhibit an optimal auditory frequency range of 2–3 kHz [38]. By comparison, the noise emitted by small, multirotor drones (sound levels reported at 57.8–81 dB, and frequencies of 60 and 150 Hz [31, 32, 42]) is likely to be audible to many taxa at low altitudes (between 5 and 10 m). However, the influence of altitude and ambient noise levels on the detectability of drones for different species remains generally understudied.

In the current study, we tested a small, commercial drone (DJI Phantom 4 Pro®) in wildlife surveys at field sites in tropical Australia: Bare Sand Island, Northern Territory (NT), Cape Domett in Western Australia (WA), and at multiple reefs across Camden Sound, WA. Collectively, these locations provide prime nesting and/or foraging habitat for sea turtles [43, 44], seabirds [45, 46], and saltwater crocodiles [47]. Yet ecological studies in these habitats are often logistically challenging, and an overall paucity of data exists regarding species in remote tropical locations of WA and the NT. Thus, the focus of the current study was to provide preliminary information that can guide the integration of drone-based studies into studies of wildlife and effective conservation resource management.

## Materials and methods

Field sites for the current study are presented in Table 1 to illustrate the types of habitats and target species encounter during drone flight trials. A 1.4-kg DJI Phantom 4 Pro® (www.dji.com) drone was used for the current study. The drone can travel up to 5 km, and each high capacity battery (5870 mAh) provides a maximum of 30 min flight time. The drone was operated using a tablet-based app (Litchi™, VC Technology Ltd.) that displayed real-time drone telemetry information (e.g. drone altitude, speed, distance, etc.). Flight records were automatically uploaded to Airdata.com. All flights were compliant with CASA regulations and conducted as part of permitted research activities by AusTurtle (NT) and Department of Biodiversity, Conservation and Attractions (WA).

**Table 1:** Study locations, habitat type, and target species observed during drone flight trials in Australia.

Study Site	Location (Lat, Long)	Habitat Type	Cb	Cs	Tb	Ts	BC
**BSI**	-12.536173130.420038	Beach, Reef	X		X		X
**CD**	-14.802778128.413889	Beach, Nearshore	X	X	X	X	
**SC**	-15.540833124.410556	Nearshore				X	
**MR**	-15.948923124.204102	Reef				X	
**TR**	-16.272721123.892815	Reef				X	

BSI, Bare Sand Island, NT, Australia; CD, Cape Domett in WA; SC, Sampson Cove in Camden Sound, WA; MR, Montgomery Reef (Yawajaba Island) in Camden Sound, WA; TR, Turtle Reef in Camden Sound, WA; Cb, crocodile basking on the beach or in the surf; Cs, crocodile swimming in nearshore waters; Tb, turtle on the beach; Ts, turtle swimming in nearshore waters; BC, bird colony.

### Sea turtles

The focus of surveys off CD, in the nearshore waters of SC, and over reef habitat was to evaluate potential behavioral responses to the presence of a drone at various altitudes between 15 and 30 m. All surveys were conducted at an initial altitude of 30 m and a constant speed of approximately 6–8 m/s. If a turtle was encountered during a survey, the behavioral response of the turtle was documented during this initial flyover at 30 m, and the drone was flown such that the aircraft did not stop and continued along the original trajectory of the survey away from the turtle. Once the drone was approximately 100 m away from where the turtle was originally observed, the aircraft was stopped, the altitude was reduced to 20 m, and the drone was flown back to where the turtle was first observed to conduct a second flyover at 20 m. If the turtle was still visible, this process was conducted again for a third flyover at 15 m. If possible, a flyover at 30, 20, and 15 m altitude was conducted for each turtle encountered, while documenting the behavioral response of the turtle during each flyover, constituting a “flight trial”. After completing a flyover at 15 m, or if the turtle was no longer visible during a flyover, the flight trial was ended, and the aircraft resumed the standard survey. Since control data (sea turtle behaviors documented when the drone was not present) was not available, behavioral responses documented in the present study of sea turtles in nearshore and reef habitats were evaluated relative to known startle responses documented in previous studies [33–36].

#### Nearshore habitat

Nine nearshore surveys were conducted off CD beach between 0730 and 1600 hrs from 5–11 August 2017. Each drone survey consisted of two straight-line transects parallel to the nesting beach, one 500 m, the other 1 km offshore. Additionally, a straight-line transect was conducted perpendicular to the nesting beach out to 2 km offshore. Nearshore surveys throughout SC were conducted on 15 August 2017, at approximately 1330 hr. Unlike surveys at CD, flights at SC were flown an altitude of 50–60 m to provide a wider field of view from the drone to locate and avoid overflying humpback whales (*Megaptera novaeangliae*) that are known to frequent the area during Austral winter. Civil Aviation Safety Authority (CASA) regulations restrict the maximum altitude of recreational drones to 120 m that conflicts with the required 500 m exclusion zone when flying near whales [48]. The flight paths at SC paralleled the coastline at approximately 300–500 m offshore.

#### Reef habitat

An interconnected network of shallow, algae-covered rocky reefs extends between the islands of Fog Bay in the NT, approximately 50 km west of Darwin [49]. Similar habitats comprise MR and TR. In the NT, two sites were surveyed between 26 June and 24 July, one south of BSI, and the other north of BSI. Thirteen surveys following the edge of the reef were conducted between 1100 and 1500 hrs.

A total of 7 surveys were conducted on MR and TR on 16–17 August, and 18 August, respectively, including one survey of MR conducted at 15 m altitude to compare the behavioral responses of sea turtles to a drone at 15 and 30 m altitude. Surveys involved two types of transect paths, one that followed the edge of the reef between exposed shallow portions of the reef and the slope, and the other traversing the region from the slope of the reef into its interior.

#### Nesting beaches

BSI and CD are important rookeries for the flatback sea turtle [43, 44, 50, 51]. Drone flights over turtles that emerged to nest during daylight were conducted at altitudes between 10 and 30 m during stages of the nesting process at which Witherington [52] indicates sea turtles are particularly susceptible to being deterred from nesting: 1) initial emergence from the sea and progression towards the dune, 2) digging a body pit, and 3) constructing an egg chamber. When a turtle was observed in any stage of the nesting process, the drone was launched from behind the primary dune and at least 500 m away from the turtle. The drone was raised to an initial altitude of 30 m, and the aircraft was flown at regular survey speed (6–8 m/s) perpendicular to the orientation of the turtle and out in front of its head to best achieve maximum visibility of the drone to each turtle. Any change in behavior or visual signs of disturbance (i.e. increased crawl speed, abrupt change in direction, abandonment of the excavated body pit or egg chamber, or return to the sea) were documented following each flyover. Drone flyovers were conducted according to the same protocols described for nearshore surveys and flyovers of least 2 consecutive stages of the nesting process were conducted for each turtle.

### Saltwater crocodiles

All survey transects were conducted according to the methods described for sea turtle surveys, from an initial altitude of 30 m and a speed of approximately 6–8 m/s. If a crocodile was encountered during a survey, the behavioral response of the crocodile to the initial flyover at 30 m was documented. The previously described protocol for conducting a flight trial during a nearshore survey was used to conduct a flight trial for each crocodile, with flyovers at 30, 20, and if possible, 10 m, to evaluate potential behavioral responses at each altitude. An additional flyover at 40 m altitude was conducted following the initial flyover at 30 m during each flight trial to refine the upper threshold drone altitude above which behavioral responses are not elicited.

#### Nearshore habitat

The abundance of saltwater crocodiles in nearshore waters was documented during 8 drone-based surveys off CD between 0730 and 1600 hrs. Surveys occurred from 5–11 August, 2017. There is a lack of control data (behaviors of crocodiles in the absence of the drone) for crocodiles in nearshore habitats and these data were therefore not available in the current study.

#### Surf zone

Drone transect surveys were conducted on BSI and on CD over saltwater crocodiles that were resting on the beach or in the surf zone. On BSI, two drone flyovers of a 2.4-meter crocodile (length measured using imagery from the drone) were conducted on 26 and 28 June 2017 at 30 and 40 m altitude, as the crocodile was resting out of the water on a sand spit. Eight transect surveys were flown parallel to the beach and over the surf zone to document crocodiles at CD between 4 and 11 August 2017. Most drone surveys were flown between 0530 and 0630 hrs when daylight was sufficient for optimal visibility of beach tracks. On 4 August 2017 a survey was conducted at 1330 hrs. Binoculars were used to observe crocodiles in the surf zone or on the beach immediately prior to each drone flight and these observations were used to evaluate the behaviors observed during each drone flight.

### Nesting birds

A sand-bank approximately 1 km in length is located southwest of BSI provided a resting location for a colony of crested terns. Eight drone surveys were conducted between 27 June and 24 July 2017, from 1200 to 1700 hrs, and at altitudes between 30 and 70 m. Flyovers were limited to 2 per day to avoid the possibility of habituation to the potential disturbance due to the drone. A flight trial began at the highest altitude being tested (e.g. 70 m) and progressed lower (e.g. 60 m) if no flushing response was elicited. If the colony took flight during flyovers, the trial was stopped. The drone was launched approximately 1 km from the resting colony of crested terns. Once the drone was within 500 m of the sand island, the drone was flown at a speed of 3–5 m/s. Although the colony of crested terns was observed using binoculars immediately prior to each drone flight from the southwestern tip of BSI, the angle of observation was not sufficient to record a quantitative description of the number of birds taking flight before the drone flight.

## Results

### Sea turtles

#### Nearshore habitat

**Fig 1:**
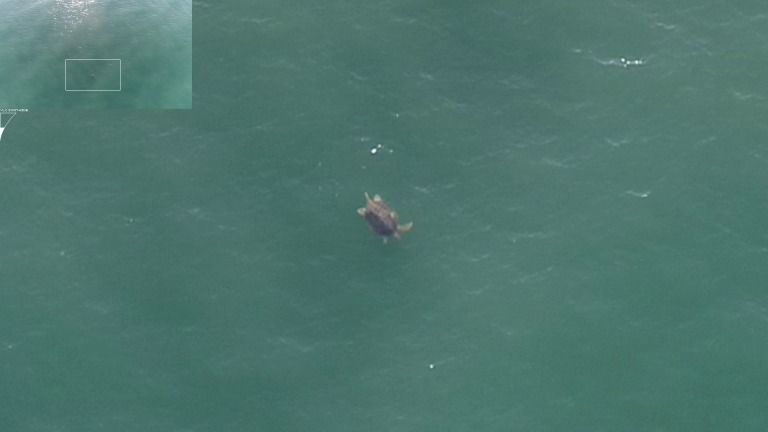
Male sea turtle observed from a drone off a nesting beach. Male flatback turtle sea turtle (*Natator depressus*) observed from a drone at an altitude of 18.6 m approximately 2 km off the nesting beach at Cape Domett, Western Australia on 6 August, 2017.

Two flatback (1 male and 1 female), 1 female green, and 1 sea turtle of unknown species and sex, were observed during drone flights over nearshore waters off CD (Fig 1 and S1 Fig). At SC, 2 flatback sea turtles were observed by drone in nearshore waters. Although the turtles encountered during flights spent relatively little time at the surface of the water (3–60 sec at CD, 10–30 sec at SC), the turtles did not exhibit avoidance behaviors (i.e. rapid diving or change in direction) in response to the presence of the drone or its shadow at or above 20 m altitude. Both sea turtles at SC submerged before drone flight trials could be conducted (S1 Table).

#### Reef habitat

**Fig 2:**
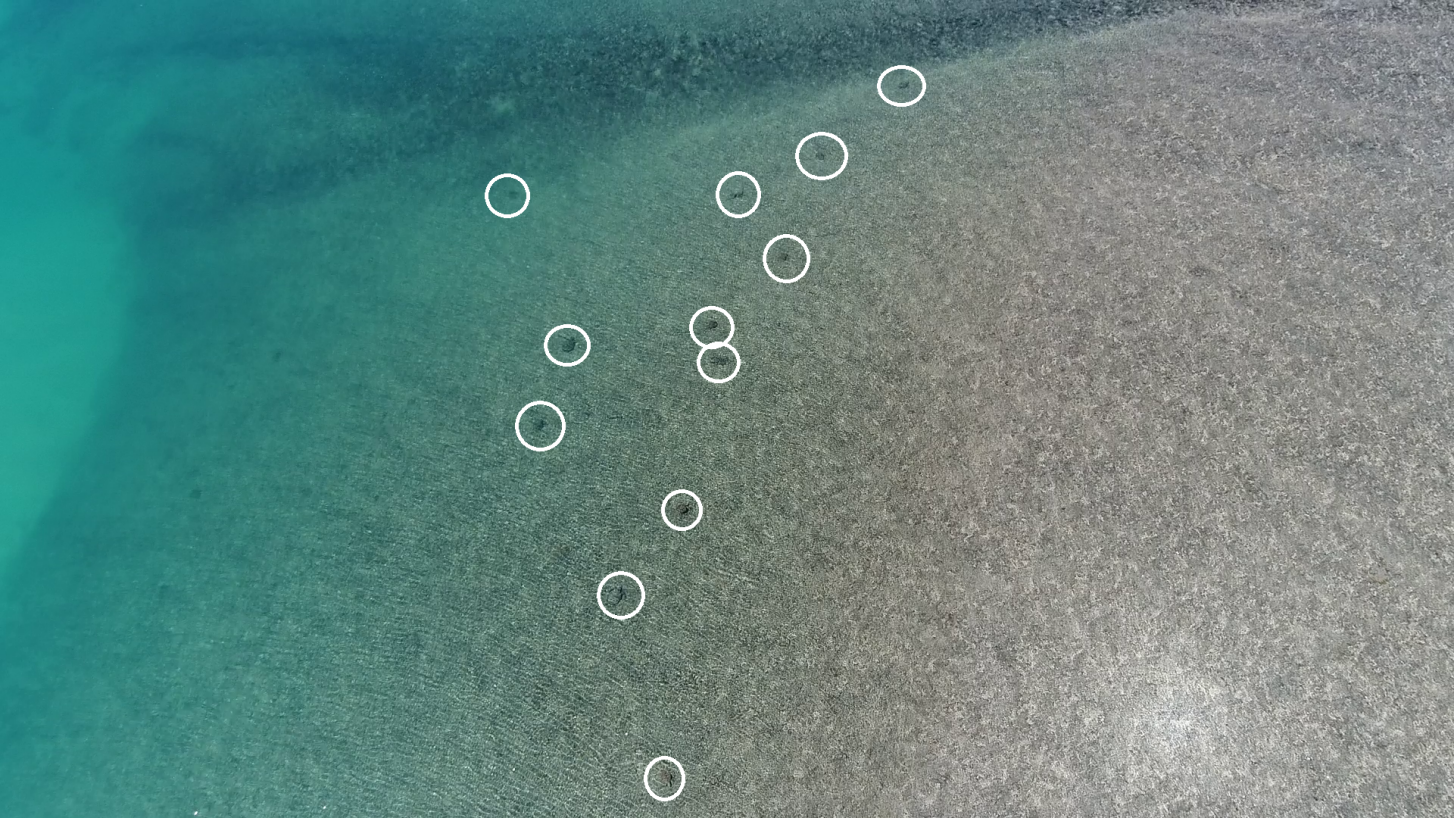
Sea turtles observed from a drone on shallow reef foraging habitat. Sea turtles observed foraging on algae-covered reef habitat from a drone altitude of 30 m at Turtle Reef, Camden Sound, Western Australia.

We surveyed the behavioral responses of multiple green and hawksbill sea turtles (>10 turtles/survey) to the drone while foraging on algae-covered rocky reef habitat at BSI, MR, and TR (Fig 2 and S2 Fig). The turtles did not exhibit avoidance behaviors (e.g. a bow wave generated by the rapid flight response) in response to the presence of the drone or its shadow at either 15 or 30 m. Individuals were completely submerged, at the surface of the water, or partially exposed while feeding (<1m water depth). The submerged sea turtles were either stationary, potentially foraging on the reef, or slowly swimming along the bottom at approximately 2 m depth or less. Turtles at the surface were encountered floating over the reef slope, or over deeper channels along the reef.

**Fig 3:**
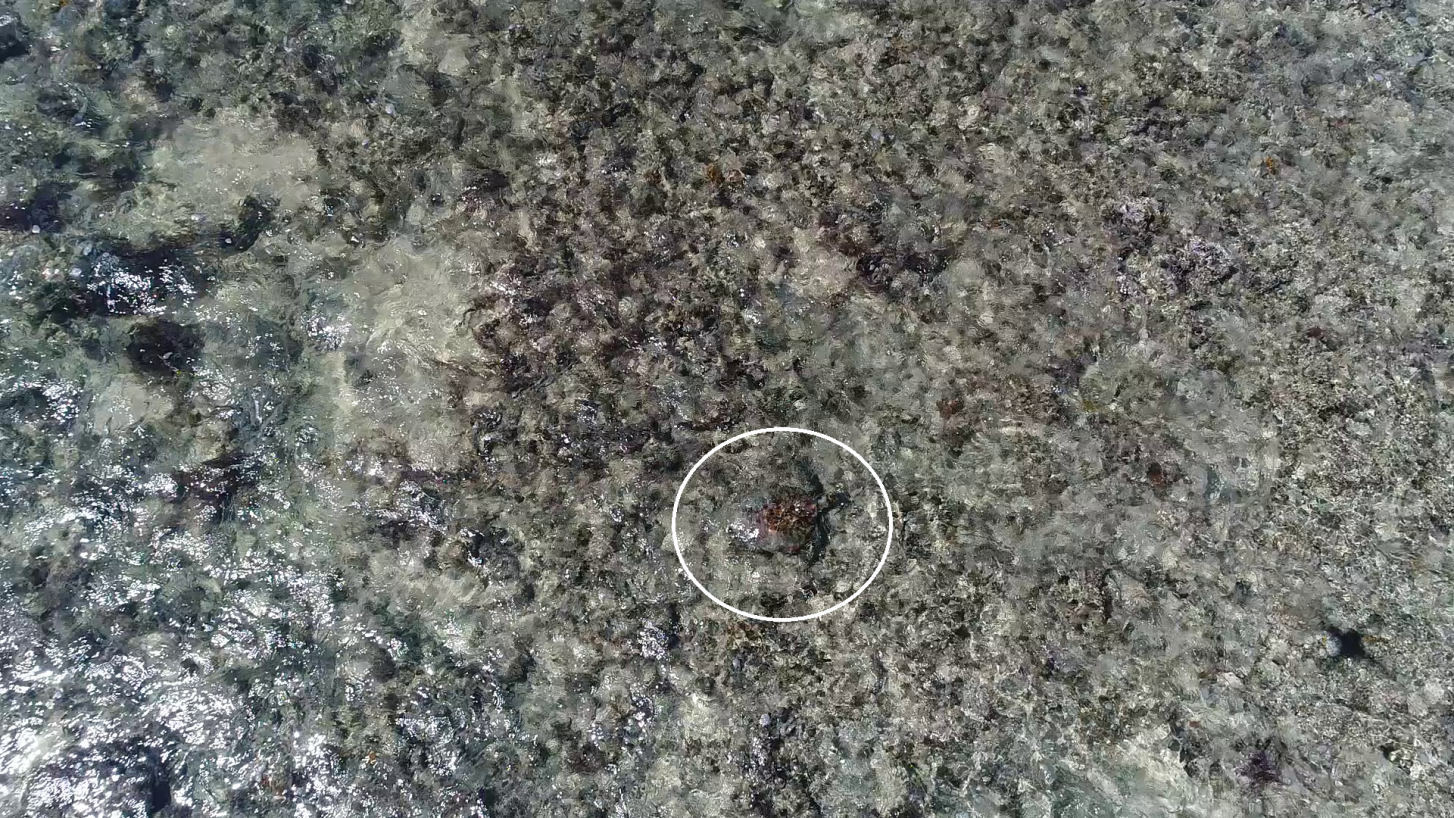
Sea turtle observed foraging on a shallow reef from a drone at low altitude. Examples of a drone being lowered to approximately 5 m over a foraging green turtle (*Chelonia mydas*) on Montgomery Reef, Camden Sound, Western Australia.

At TR and MR, the drone altitude for two flights was reduced from 30 m to 9 m and 5 m, respectively. In both instances, sea turtles were foraging in less than 2 m of water. At MR, the green sea turtle was in less than 0.5 m depth of water and displayed no response to the presence of the drone, despite a shadow cast in front of the turtle (Fig 3). At TR, a hawksbill sea turtle was observed slowly swimming over the reef in 1–2 m depth of water (S3 Fig). In the presence of the drone at 9 m altitude, the individual increased the force of flipper strokes, potentially to accelerate to deeper water before slowing and turning around. This behavior could be classified as a minor behavioral response to a drone flying at low altitude. However, rapid avoidance or major evasive responses were not observed during this trial.

#### Nesting beach

**Fig 4:**
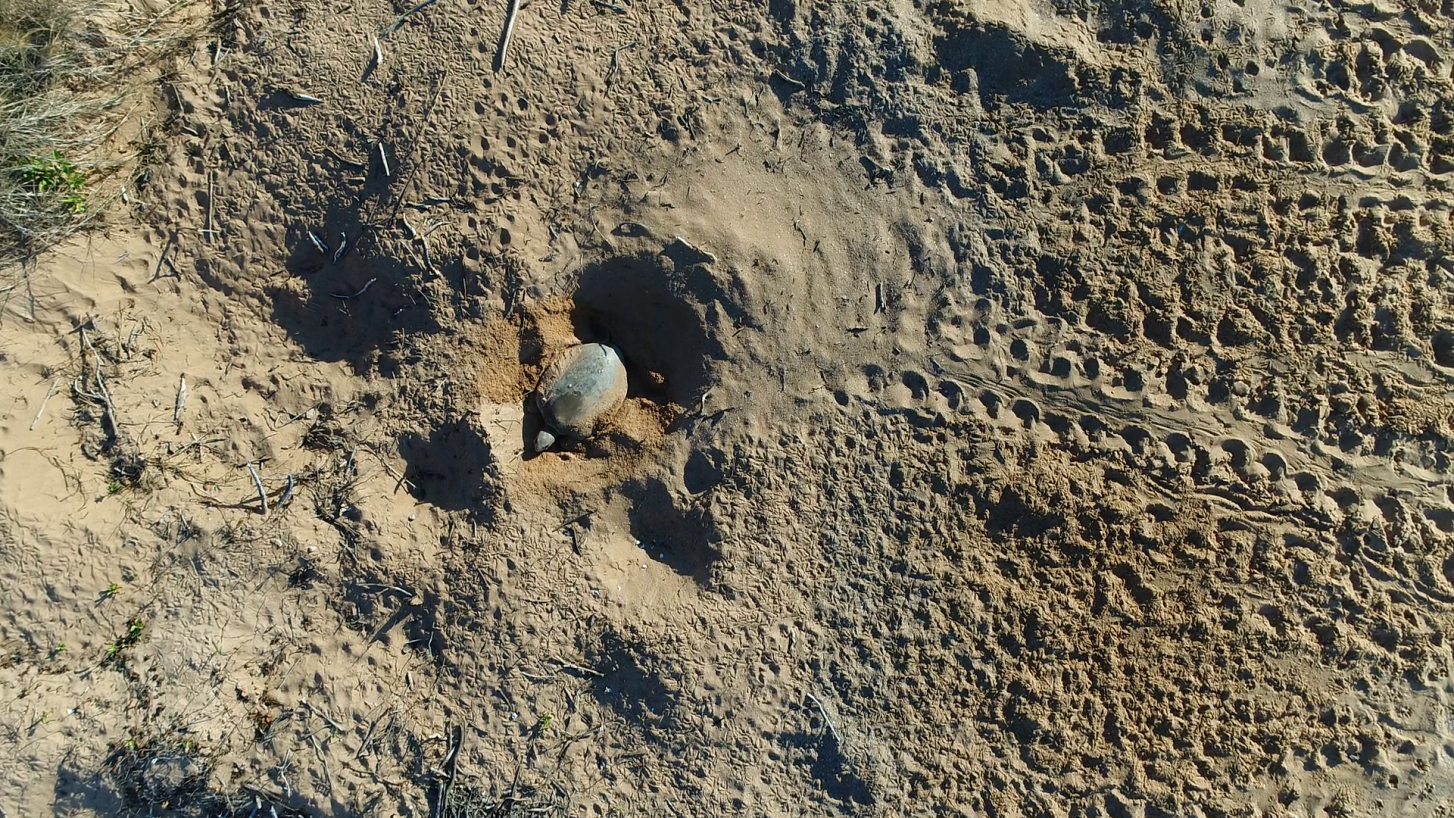
Behavioral response of a sea turtle during the nesting process observed from a drone at low altitude. Testing the behavioral response of a flatback turtle (*Natator depressus*) to the presence of a drone at 10 m altitude during the nesting process at Cape Domett, Western Australia.

At BSI, a female flatback sea turtle emerged to nest on 25 July at 1900 hr. At CD, 5 female flatback sea turtles emerged to nest from 5 to 9 August between 1600 and 1700 hrs. No disruption or abandonment of the nesting attempt was observed for any of the turtles encountered at any altitude. Collectively, the stages of nesting during which a female turtle is likely to be disturbed, (i.e. emerging from the sea, and digging a body pit and egg chamber) were examined for signs of drone disturbance at altitudes between 10 and 40 m (Fig 4).

### Saltwater crocodiles

Eleven drone surveys were conducted at BSI and CD, resulting in 31 crocodile sightings. It is possible that the same individuals were observed on multiple surveys over the study period, given the tendency of crocodiles to return to established core activity areas [47]. However, individual identity could not be verified in the present study and each crocodile observed was treated as a new sighting.

#### Nearshore habitat

Eighteen of the 31 crocodile sightings in the present study were observed swimming in nearshore habitat at CD. The crocodiles did not exhibit avoidance behaviors (e.g. movement of the head or body) in response to the presence of the drone or its shadow at 30 m or above. Initial signs of disturbance were observed at a maximum drone altitude of 30 m and involved one or more lateral movements of the head. Drone flight trials conducted at 20 m altitude elicited more pronounced lateral head movements, and flight trials conducted at 10 m altitude resulted in crocodile submergence (S1 File).

#### Surf zone

At BSI, one crocodile was observed basking on the sand during each survey. One to 4 crocodiles were observed during each drone survey at CD. Signs of crocodile disturbance from the drone when basking on the sand included minor to substantial lateral head movements and/or retreat to deeper water. However, in contrast to BSI, crocodiles at CD were only observed basking on the sand on the initial day of surveys (4 August), after which all crocodiles were observed resting in the surf or swimming in nearshore waters. Crocodiles were observed basking motionless on the sand or in the surf zone immediately prior to each drone flight.

**Fig 5:**
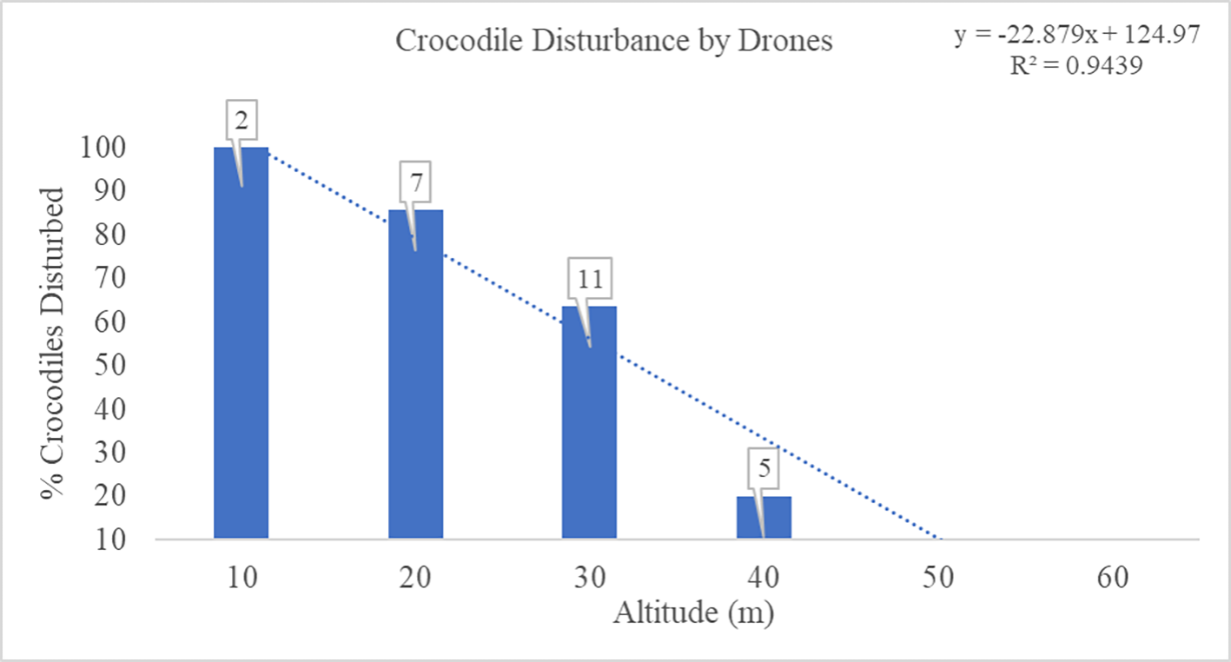
Crocodiles disturbed by a drone at various altitudes. Percentage of crocodiles (*Crocodylus posorus*) observed during drone-based surveys Bare Sand Island, Northern Territory, and Cape Domett, Western Australia, that were disturbed by the presence of a drone at various altitudes. Results are based on a total of 31 crocodile sightings over 10 surveys observed either resting on the beach, in the surf, or actively swimming on the surface of nearshore waters between the surf and approximately 300 m off the nesting beach. The numbers of total crocodiles sampled for each altitude are show in boxes above the bars.

**Fig 6:**
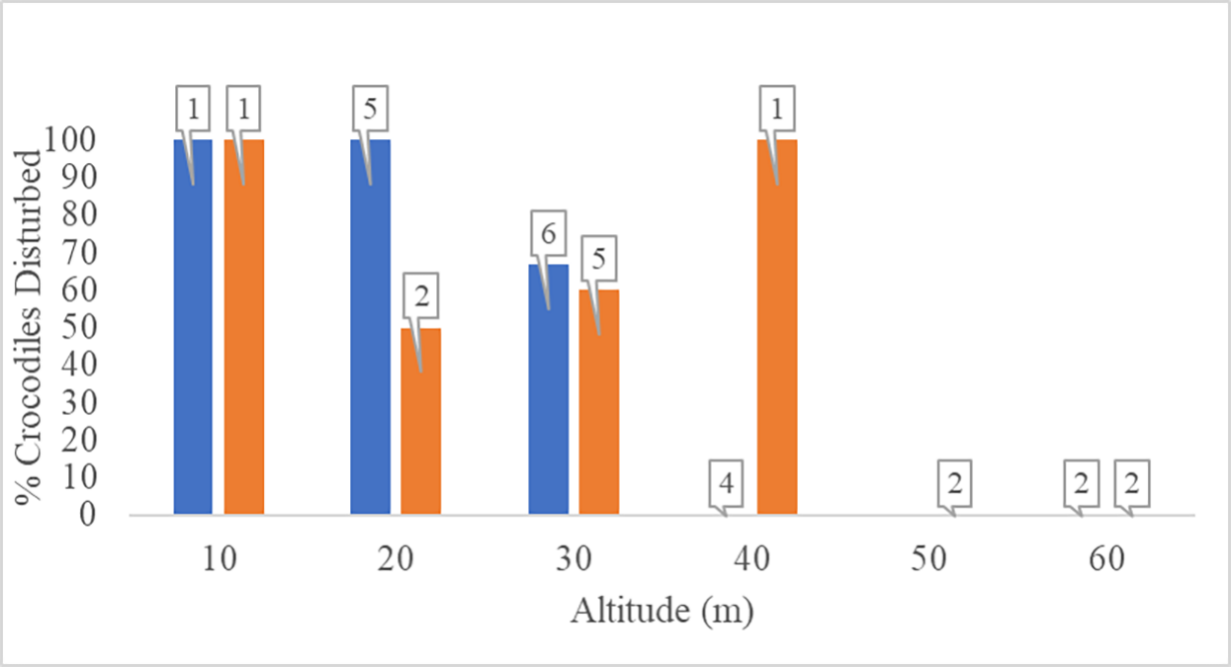
Drone disturbance to crocodiles by habitat. The percentage of crocodiles (*Crocodylus posorus*) that were disturbed by the presence of a drone while resting on the beach or in the surf (orange bars), or actively swimming in nearshore waters (blue bars) at Bare Sand Island, Northern Territory, and Cape Domett, Western Australia. The numbers of total crocodiles sampled for each altitude are show in boxes above the bars.

Collectively, drone surveys of crocodiles at BSI and CD suggest that adult and sub-adult crocodiles basking on the sand or swimming in nearshore waters are disturbed by drones when flying below approximately 50 m in altitude (Figs 5 and 6). All trials conducted at 10 m altitude caused rapid head movements, after which crocodiles either submerged or retreated to deeper water (S2 File).

### Nesting birds

**Fig 7:**
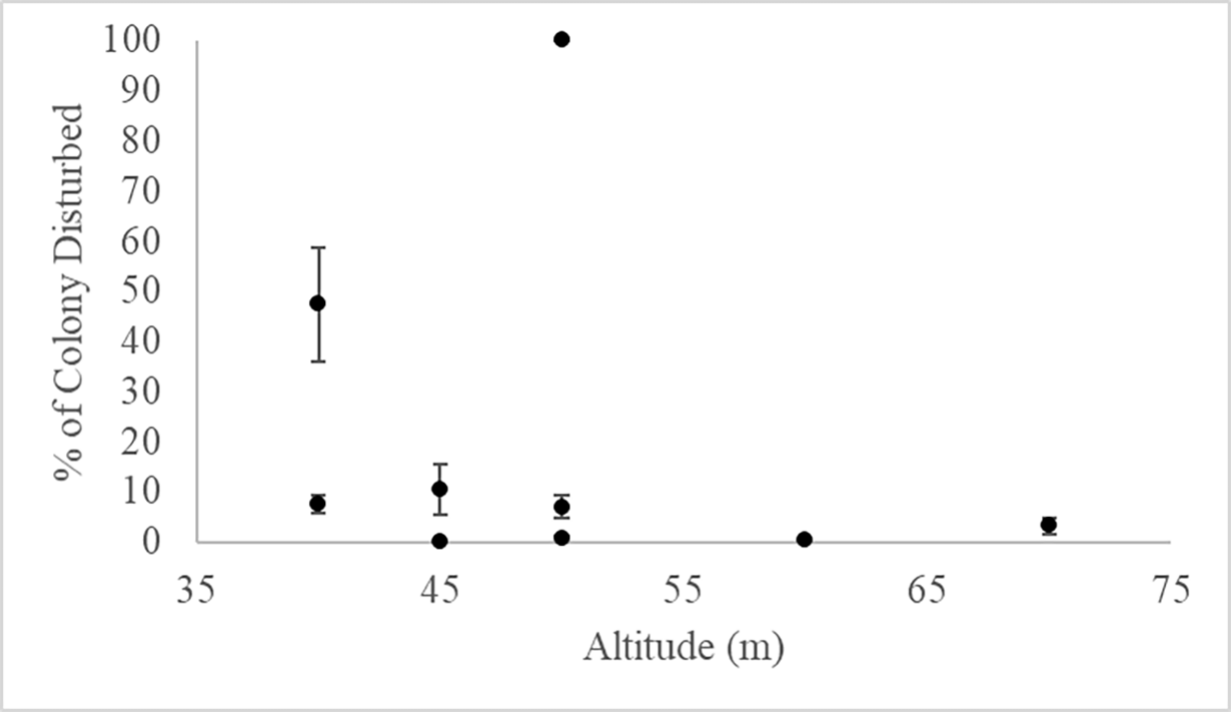
Crested terns disturbed by a drone at various altitudes. The percentage of crested terns (*Thalasseus bergii*) observed on the sand-bank to the southwest of Bare Sand Island, NT that took flight as a drone passed overhead at various altitudes. Although trials at altitudes lower than 40 m were possible ethical considerations and research permits prevented flyovers at lower altitudes.

The mean size of the crested tern colony on the sand-bank throughout the 8 drone surveys was 153 birds (range = 19–334 birds) (Table 2). Flight trials indicated that the crested tern colony was generally disturbed by a drone flying below 60 m altitude (Fig 7). Observed disturbance behaviors consisted of increased vigilance (i.e. raised wings and multiple, lateral head movements) and flushing of one to many birds in the colony.

**Table 2:** Drone flight trials over a colony of crested terns on a sand-bank southwest of BSI from 27 June to 24 July, 2017.

Date	Time	Altitude	Tide	# Birds that took flight	Total # of birds	% Disturbed
**3-Jul**	1700	70	Falling	4	121	3.3%
**27-Jun**	1328	60	Falling	1	334	0.3%
**28-Jun**	1430	50	Falling	246	246	100.0%
**4-Jul**	1215	50	Rising	9	128	7.0%
**6-Jul**	1430	50	High	1	172	0.6%
**9-Jul**	1432	45	Rising	0	112	0.0%
**24-Jul**	1515	45	Rising	4	38	10.5%
**6-Jul**	1430	40	High	16	211	7.6%
**24-Jul**	1515	40	Rising	9	19	47.4%

## Discussion

Collectively, the present approach threshold study demonstrates that the altitude for eliciting disturbance when using a drone varies by taxa. Factors that contribute to the specific disturbance threshold for a given species include the ability of a species to detect the visual disturbance of drones and the unique sound signature of a particular drone, the degree to which drone disturbance is associated with a threatening stimulus (a predator), and the ambient noise level of a given habitat. Although sound levels emitted by the DJI Phantom 4 Pro® used in the current study have not been quantified, it is likely similar to noise levels reported for other drones of the same size class used in previous studies (57.8–81 dB, and frequencies of 60 and 150 Hz) [12, 31, 42]. Characterizing the noise emitted by multirotor drones is challenging since sounds emitted by the aircraft change in response to wind gusts while hovering, and to operator controls during flight [42]. At 30 m altitude, these sound levels may be lost against background noise, such as in a penguin colony [32], or may be undetectable at 1 m below the surface of the water when the drone is at approximately 10 m altitude [53]. Coupling these findings with known auditory capabilities of several species of cetaceans, penguins and pinnipeds, it is likely these marine species would be unable to detect small drones [31, 32]. Each unique sound profile presents a distinct auditory stimulus to target species and can elicit different types and intensities of animal behaviors in response.

Based on previous studies of sea turtles, the maximum range of auditory sensitivity for *C*. *mydas* and *C*. *caretta* is from 100 to 1000 Hz in water, and although this range can vary with taxa and age class, other species of sea turtle are likely to exhibit similar capabilities [39, 40, 54]. Thus, it is possible sea turtles can detect the noise levels reported in previous studies for small, commercial drones at altitudes between 5 and 10 m (50–200 Hz, 57.8–80 dB; [12, 31, 32, 42]). This is especially likely without higher levels of background noise, such as water rushing off the reef flat, to mask the sound from the drone. One note of caution in our study is that 4 of the 6 sea turtles encountered in nearshore waters exhibited relatively short surface intervals (3–60 seconds), which could be interpreted as a potential behavioral response to drone disturbance. However, the degree to which sound levels emitted by commercial drones are influenced by higher flight altitudes (e.g. 15 m or higher) and background noise remains largely undetermined [31, 32].

The auditory capabilities of crocodiles are concentrated at low frequencies and coupled to a diverse array of vocalizations that aid in group coordination, mating, territorial defense, and maternal care [41, 55, 56]. Based on previously reported sound levels for small, commercial drones at altitudes between 5 and 10 m, and our results from the current study, it is possible crocodiles can visually detect and/or hear small drones at low altitudes. Further, our preliminary findings suggest that specific activities (i.e. basking on the beach, in the surf, or actively swimming in nearshore waters) may influence the threshold altitude above which a drone can be used without eliciting behavioral changes from crocodiles (Fig 6).

The results from the present study suggest that using drones to study colonies of *T*. *bergii* requires a higher altitude approach (> 60 m) than the other species we investigated to avoid disturbance. The auditory capabilities reported for birds (optimal frequencies between 2–3 kHz), paired with the sound levels emitted by small commercial drones (50–200 Hz, 57.8–80 dB), suggests that the noise emitted by drones may not be audible to colonies of *T*. *bergii*, but that the disturbance may be due to other factors (e.g. visual disturbance). A previous study supports our conclusion that this species is not disturbed by the presence of a drone above 60 m altitude [6]. Nevertheless, studies are needed that decouple the auditory and visual components of drone disturbance to evaluate behavioral disturbance thresholds for birds.

An additional concern when evaluating the potential for drone disturbance is whether a given species is startled by visual detection of the drone and/or its shadow. If sea turtles could visually detect and were disturbed by the drone at 15–30 m altitude, we would have expected to see rapid avoidance behavior (e.g. submergence behavior, swimming towards deeper water). For adult sea turtles at nearshore habitat and juveniles foraging on reefs, our drone approached many of the individuals from either the front or within the peripheral field of view of the sea turtle and cast a shadow that was potentially visible to these individuals. However, no sea turtles exhibited evasive behaviors, suggesting that individuals either are unable to visually detect a drone at 15–30 m altitude, or did not perceive the drone/its shadow as a threat. Thus, it is possible that drone shadows are not impacting the behavior of sea turtles. These results suggest that altitudes above 15 m are adequate for providing high resolution imagery of sea turtles in nearshore waters and shallow reef habitat and documenting natural sea turtle behaviors. Further investigation is needed to characterize the behaviors of different sea turtle species in response to the potential visual disturbance of a drone at low altitudes and/or its shadow.

The current study supports the use of drone technology in studies of marine species in a variety of habitats. Our findings indicate that operating a drone at or above 20 m altitude is a non-invasive protocol for studying sea turtles in nearshore waters off nesting beaches. Likewise, operating a drone at or above 15 m altitude in shallow reef habitats may be an optimal method for use in behavioral studies of sea turtles. At sea turtle nesting habitats, the results from the current study were consistent across multiple locations and populations and suggest that the nesting processes of flatback sea turtles are not disrupted by drones at or above 10 m altitude. If sea turtles can detect the noise emitted from small drones at low altitudes (5-10m), the drone did not provide a perceived threatening stimulus sufficient to change nesting behavior or cause abandonment of a nesting attempt. Collectively, our findings suggest that drones can be used to study sea turtles at low altitudes (from 10–30 m) without disturbing individuals, but that the threshold for disturbance for each species depends on habitat and environmental conditions. Drone technology may therefore be an optimal tool for eliminating human observer presence, a known factor in sea turtle disturbance [52], while studying nesting processes or monitoring nesting activity on beaches.

Drone surveys of saltwater crocodiles resting on sea turtle nesting beaches and resting or swimming in nearshore waters suggests that operating a small drone at lower altitudes (10–30 m) to study crocodile behaviors can cause behavioral disturbance. Previous studies have used a variety of drones at altitudes of 100 to 300 m to study crocodilians with no indication of disturbance to individuals [57–59]. However, these altitudes may be insufficient for mapping or behavioral studies that require imagery in greater detail and higher resolution, and therefore necessitate lower altitude surveys. Collectively, video imagery from the current study suggests crocodiles can detect visual and/or auditory disturbance from a small drone. However, studies evaluating the visual and auditory capabilities of crocodilians, and quantifying the disturbance caused by commonly-used drones are areas of need in conservation management research.

At Raine Island National Park (RINP), Queensland, preliminary data suggests that other avian species are even more sensitive to drone disturbance than *T*. *bergii* [60]. Official guidance for drone use within RINP indicates that drone altitudes of 80 and 120 m, respectively, are required to avoid disturbing brown booby (*Sula leucogaster*) and common noddy (*Anous stolidus*) birds. These requirements suggest that drone disturbance may be species-specific, and that different avian taxa exhibit different behavioral disturbance threshold altitudes. Such thresholds for target species should be determined prior to initiating drone-based biological studies. Future studies of *T*. *bergii* should incorporate other factors, such as environmental conditions, time of day, and reproductive status, to determine how these factors may influence behavioral reactions of *T*. *bergii* to drones. Previous studies of crested tern flight patterns indicate that non-breeding individuals regularly take flight while breeding individuals typically remained grounded during the breeding season (March–July) [45, 61]. The flight trials in the current study were conducted between June and July 2017, which falls within the known breeding season for *T*. *bergii* in the NT [45]. Thus, despite the lack of control data (number of individuals that take flight prior to a drone flight) for comparison in the current study, flights when a large portion of individuals took flight could represent a startle response due to the presence of the drone rather than normal flight movements. However, breeding colonies in this region have been reported to number in the thousands to hundreds of thousands, and the average colony size reported in the current study was a few hundred individuals. It is possible that the group of *T*. *bergii* observed by drone on the sand bank represents a relatively small portion of individuals from a nearby larger breeding colony. A comparison of the behavioral disturbance thresholds of non-breeding with breeding colonies could provide insights on whether reproductive status influences behavioral responses to drones.

An important consideration for the current study is that the results for species observed in the water are representative of individuals located within the depth of detection from the drone, which is directly influenced by water clarity. Therefore, it is possible that some individuals could have been obscured by reduced water clarity at some study sites. Additional studies and larger sample sizes are necessary to account for individual variation in behavioral responses to drone disturbance, as well as the influence of environmental factors such as water clarity and habitat type on results. A simple power analysis suggests a larger sample size (>52 individual sightings) would provide the ability to detect a medium effect of drone disturbance on the behavioral responses of a given species as a factor of drone altitude. Future studies should also aim to obtain behavioral control data for each species (behaviors displayed in the absence of a potentially disturbing stimulus, such as the presence of a drone or human observer). Collectively, future drone-based behavioral studies should incorporate these considerations as factors in their experimental design.

We used the same drone coupled with consistent protocols to evaluate behavioral responses of multiple species to drones throughout a range of habitats across northwestern, tropical Australia. It is likely that different drones and flight patterns may elicit different behavioral responses for the species evaluated. However, with drone type and flight pattern held relatively constant, the differences in threshold altitude that elicits disturbance behaviors are indicative of fundamental differences between the species. Of note, these differences in behavioral responses to drones may be founded in the basic ecology of each species and the level of response to drones may relate to the degree to which each species associates drone disturbance with the threat of a predator [12]. Regardless, the findings characterize important threshold altitudes above which the behaviors of target species do not appear to change.

## Conclusions

Drones are rapidly revolutionizing the observational and monitoring capabilities of scientists working in remote habitats where survey locations are often logistically challenging or dangerous to access. However, without first quantifying the impact of drones on wildlife, the benefit of minimizing observer presence may be diminished. We have demonstrated that a variety of disturbance thresholds exist for the suite of species that may occur within a single habitat. In establishing optimal drone-use protocols, resource managers are challenged with balancing the quality and type of data needed, with the level of disturbance inflicted upon a variety of species. The current study provides preliminary information to address these concerns and highlights promising directions for future research in this advancing field.

## Supporting information

S1 File Behavioral response of a saltwater crocodile to a drone flyover at 20 m altitude in nearshore waters off CD beach. (MP4)

S2 File Behavioral response of a saltwater crocodile to a drone flyover at 30 m altitude while resting on the sand at BSI. (MP4)

S1 Fig Sea turtle observed from a drone off a nesting beach. Female flatback sea turtle (Natator depressus) observed from a drone approximately 250 m off the nesting beach at Cape Domett, Western Australia. Individual was observed at an altitude of 30 m on 5 August, 2017.
(TIF)

S2 Fig Sea turtles observed from a drone foraging in shallow reef habitat. Sea turtles observed foraging on algae-covered reef habitat from a drone altitude of 30 m at (A) Bare Sand Island, Northern Territory, and (B) Montgomery Reef, Camden Sound, Western Australia.
(TIF)

S3 Fig Sea turtle observed foraging on a shallow reef from a drone at low altitude. Examples of a drone being lowered to approximately 9 m over a foraging hawksbill sea turtle (*Eretmochelys imbricata*) at Turtle Reef, Camden Sound, Western Australia.
(TIF)

S1 Table Sea turtles observed during drone surveys of nearshore habitat at CD and SC between 5 and 15 August, 2017. (DOCX)
