# Supplementary figures and images for: Measuring behavioral responses of sea turtles, saltwater crocodiles, and crested terns to drone disturbance to define ethical operating thresholds
Source: PLoS One. 2018 Mar 21;13(3):e0194460. doi: 10.1371/journal.pone.0194460 (PMC5862495; doi:10.1371/journal.pone.0194460)

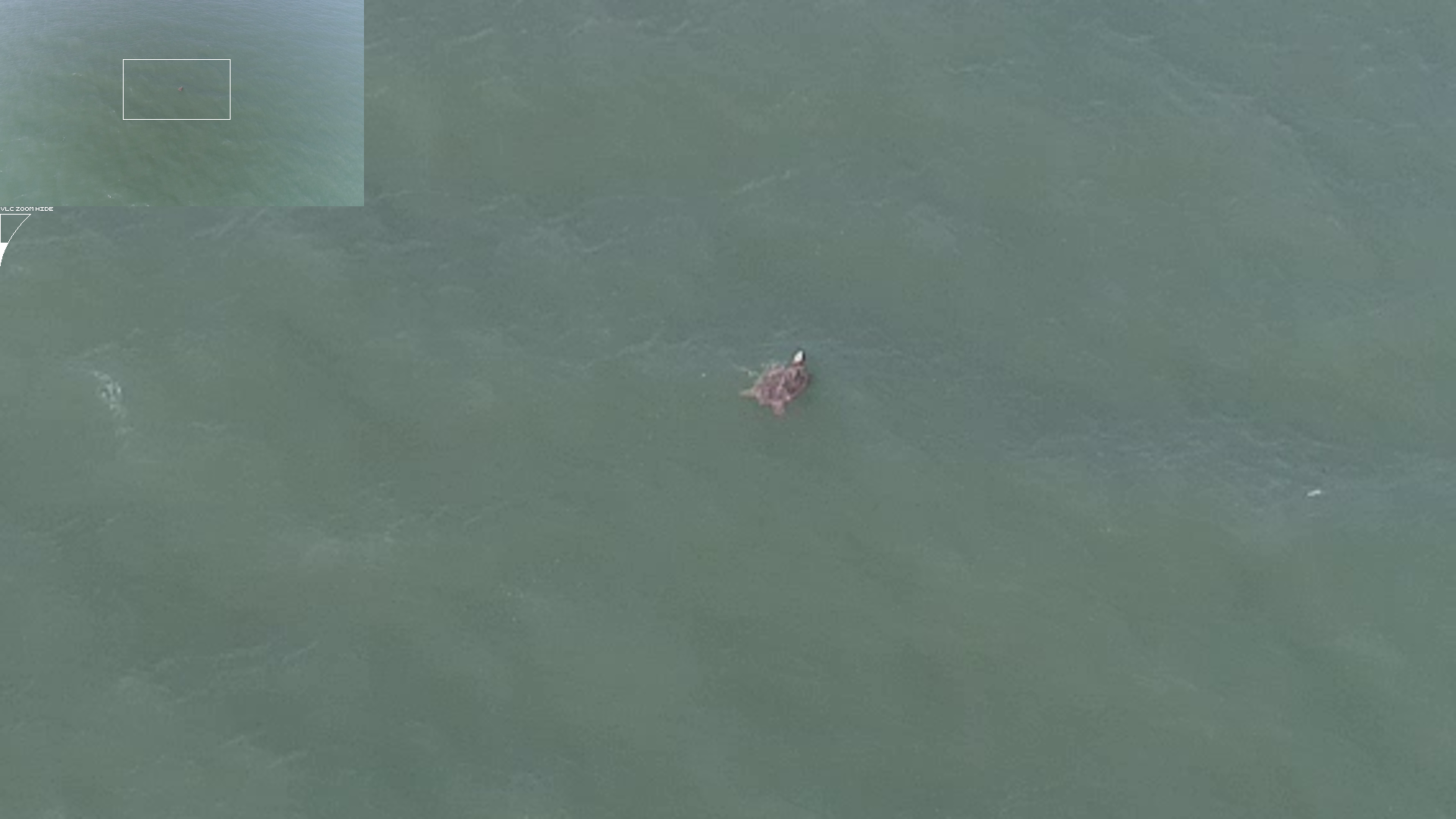

Supplement: S1 Fig — Female flatback sea turtle (Natator depressus) observed from a drone approximately 250 m off the nesting beach at Cape Domett, Western Australia. Individual was observed at an altitude of 30 m on 5 August, 2017. (TIF) [file pone.0194460.s003.tif]

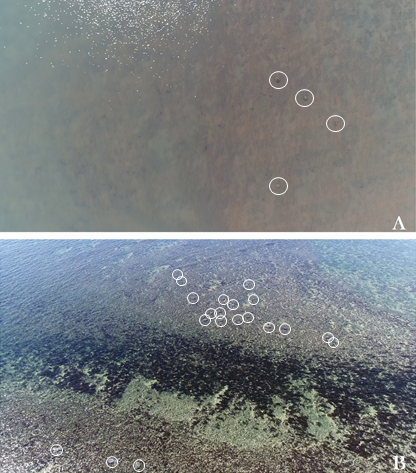

Supplement: S2 Fig — Sea turtles observed foraging on algae-covered reef habitat from a drone altitude of 30 m at (A) Bare Sand Island, Northern Territory, and (B) Montgomery Reef, Camden Sound, Western Australia. (TIF) [file pone.0194460.s004.tif]

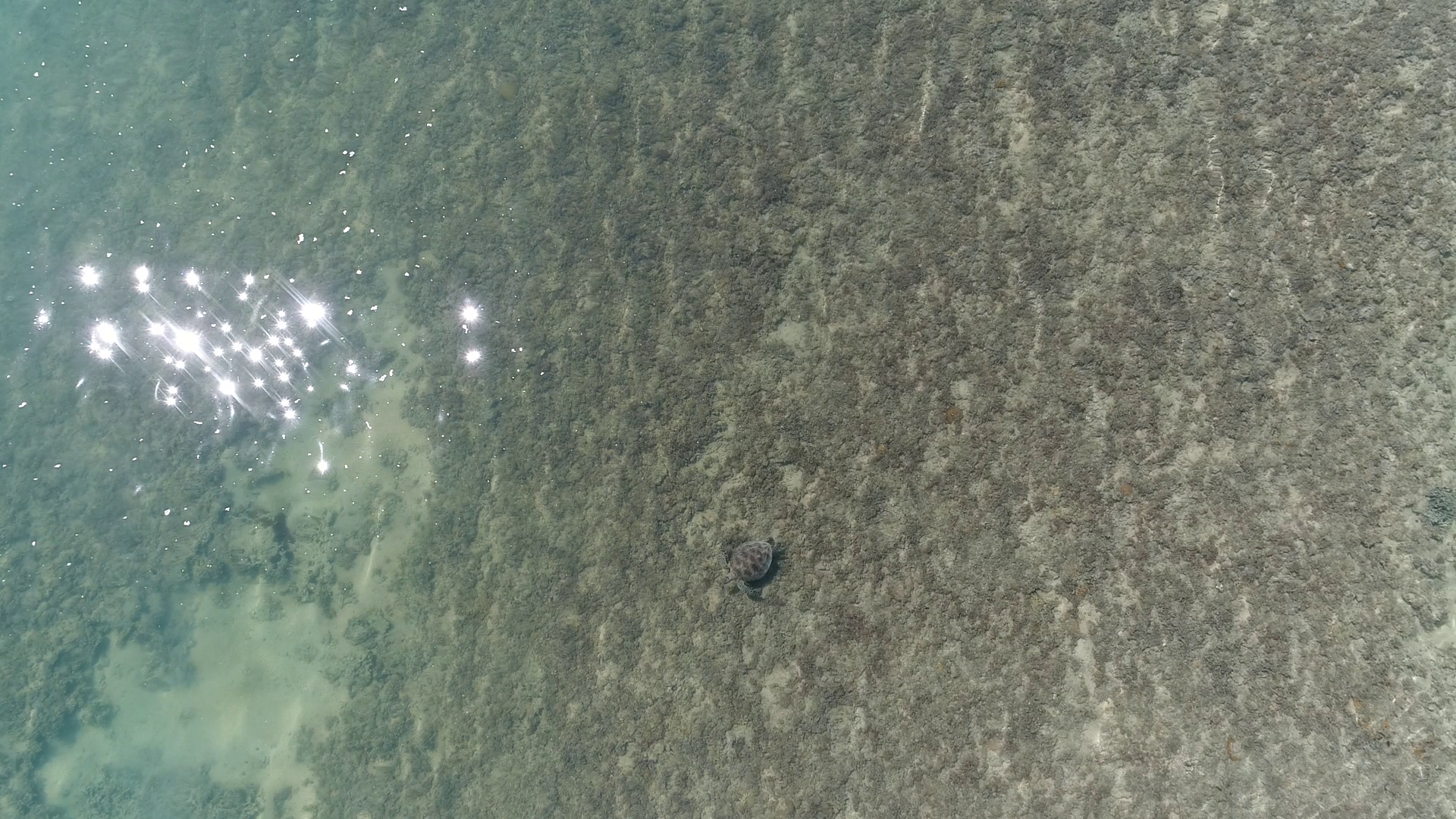

Supplement: S3 Fig — Examples of a drone being lowered to approximately 9 m over a foraging hawksbill sea turtle (Eretmochelys imbricata) at Turtle Reef, Camden Sound, Western Australia. (TIF) [file pone.0194460.s005.tif]
