# Supplementary material for: Measuring behavioral responses of sea turtles, saltwater crocodiles, and crested terns to drone disturbance to define ethical operating thresholds
Source: PLoS One. 2018 Mar 21;13(3):e0194460. doi: 10.1371/journal.pone.0194460 (PMC5862495; doi:10.1371/journal.pone.0194460)
Supplement: S1 Table — (DOCX) [file pone.0194460.s006.docx]

**S1 Table.** **Sea turtles observed during drone surveys of nearshore habitat at CD and SC between 5 and 15 August, 2017.**

| **Date** | **Loc** | **Time (hrs)** | **Alt (m)** | **TO-D** | **Dist (m)** | **ST (sec)** | **Species** | **Sex** |
| --- | --- | --- | --- | --- | --- | --- | --- | --- |
| **5-Aug** | CD | 1400 | 30 | 90° periphery | 963 | 3 | N/A | NA |
| **5-Aug** | CD | 1400 | 30 | 150° rear-right | 250 | 7 | Nd | F |
| **6-Aug** | CD | 1046 | 30 | 180° head-on | 435 | 73 | Cm? | F |
| **6-Aug** | CD | 1600 | 18.6 | 180° head-on | 1900 | 40 | Nd | M |
| **15-Aug** | SC | 1330 | 60 | 180° head-on | 305 | 26 | Nd | NA |
| **15-Aug** | SC | 1330 | 60 | 110° rear-right | 40 | 10 | Nd | NA |

Loc, location; Alt, altitude; TO-D, turtle orientation with respect to the drone; Dist, distance offshore; ST, surface time of the sea turtle; Nd, *Natator depressus*; Cm, *Chelonia mydas*.
